# Supplementary material for: Antioxidative Potential and Ameliorative Effects of Rice Bran Fermented with Lactobacillus against High-Fat Diet-Induced Oxidative Stress in Mice
Source: Antioxidants (Basel). 2024 May 24;13(6):639. doi: 10.3390/antiox13060639 (PMC11201030; doi:10.3390/antiox13060639)
Supplement: Supplementary file 1 [file antioxidants-13-00639-s001.zip › antioxidants-2977013-supplementary.pdf]

Table S1 HPLC gradient elution program

| **Time（min）** | **Solvent A（%）** | **Solvent B（%）** |
| --- | --- | --- |
| 0.0 | 0.0 | 100.0 |
| 5.0 | 15.0 | 85.0 |
| 10.0 | 35.0 | 65.0 |
| 15.0 | 65.0 | 35.0 |
| 20.0 | 85.0 | 15.0 |
| 22.0 | 95 | 5 |

Table note: solvent A is acetonitrile, solvent B is water.

Table S2 Primer sequences used for liver tissue RT-PCR in mice.

| **Gene** | **Forward primer** | **Reverse primer** |
| --- | --- | --- |
| β-actin | ACTGCCGCATCCTCTTCCTC | CTCCTGCTTGCTGATCCACATC |
| Plin5 | CAACTTTCCTGCCCGTCAAAG | GATCACCGGACATTCTGCTGT |
| Nrf2 | CCAGCTACTCCCAGGTTGCC | GGGATATCCAGGGCAAGCGA |
| NQO1 | CGACAACGGTCCTTTCCAGA | GCAGGATGCCACTCTGAATC |
| Sod2 | TGGAGAACCCAAAGGAGAGTTG | GCCTGAACCTTGGACTCCC |
| Gclc | TGCTCAAGTGGGGTGACGA | GGTCGGATGGTTGGGGTT |
| HO1 | CCCCACCAAGTTCAAACAGC | AGCTCCTCAAACAGCTCAATGT |

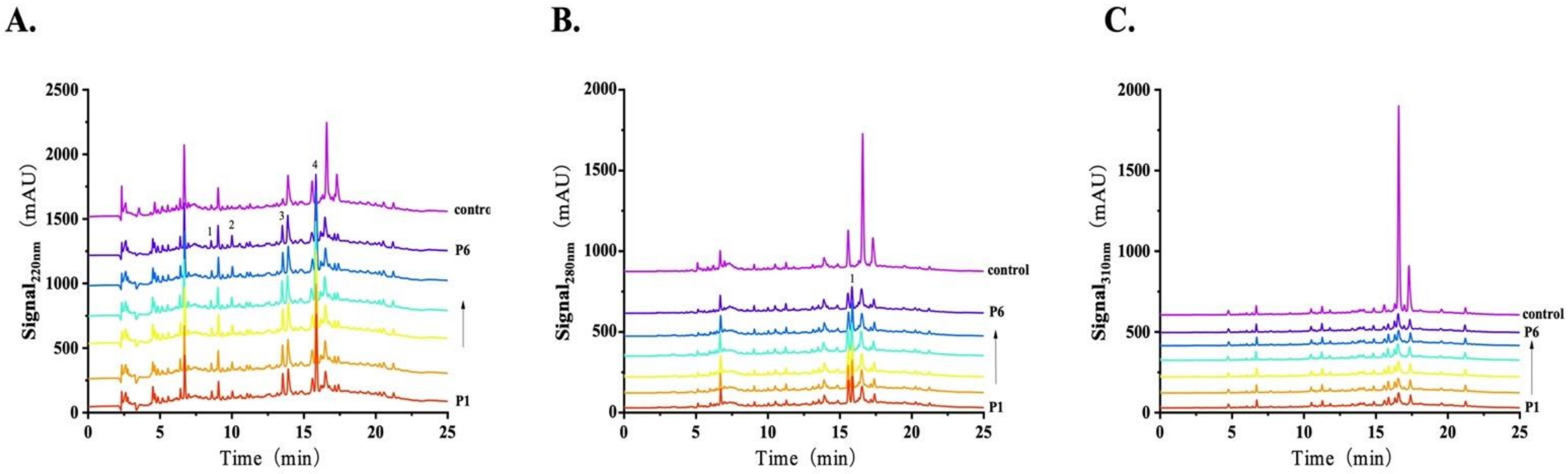


Figure S1 The HPLC chromatograms obtained from the analysis of fermented products from different batches. a-c depicts absorption peak chromatograms obtained from samples of batches P1-P6 at wavelengths of 220 nm, 280 nm, and 310 nm.

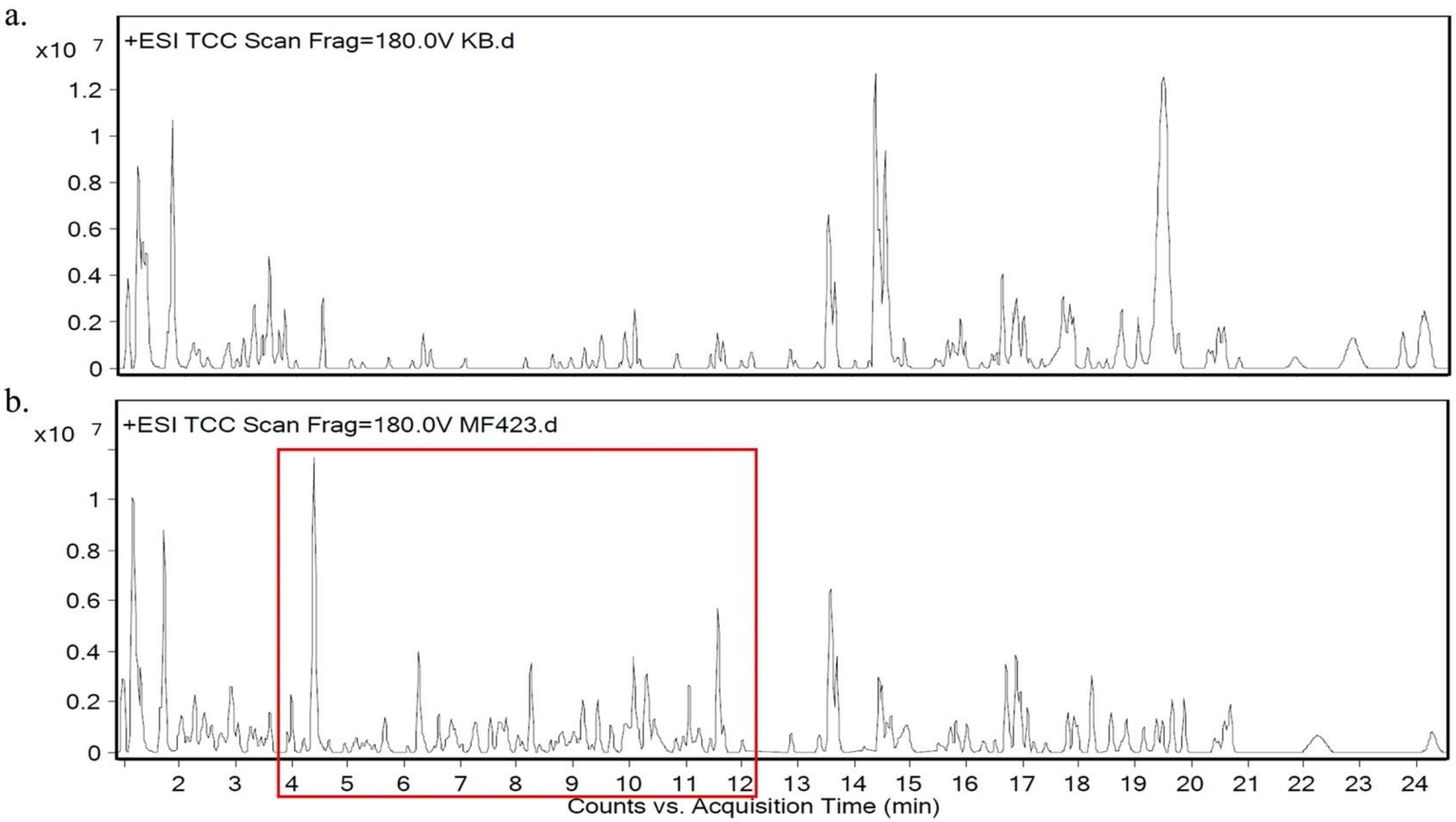


Figure S2. UHPLC-MS results of FLRB. The postfermentation peaks (b) showed significant changes in substance composition compared with the prefermentation peaks (a) in the time range of 4–12 min.

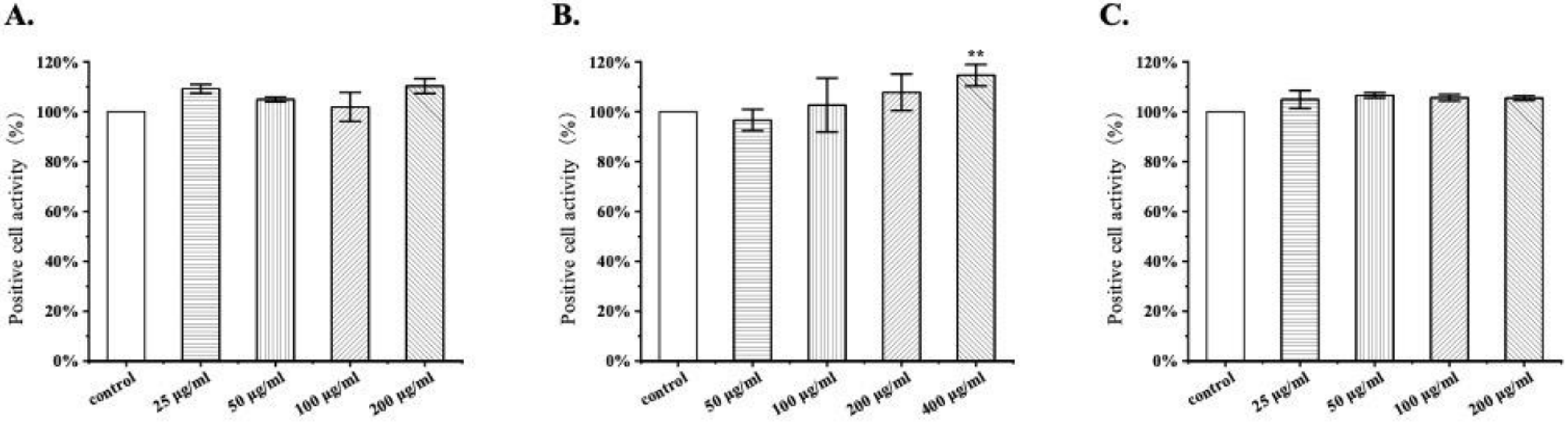


Figure S3 The effects of different concentration of FLRB on HUVECs (a), HDFs (b), HaCaT (c) cell viability were measured by MTT assay. Values were expressed as the mean ± SD (n=3).

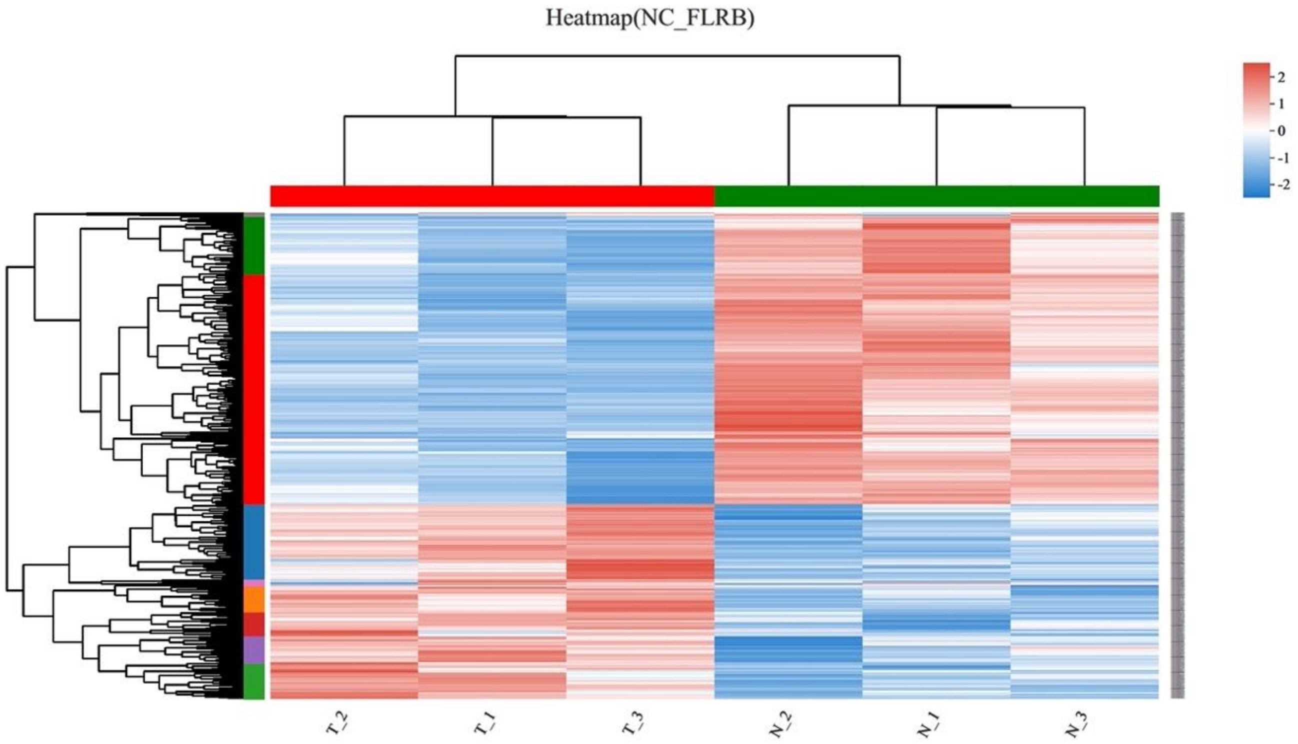


Figure S4 Heat map of differentially expressed gene. In the diagram, N_1, N_2, N_3 represent the model group, T_1, T_2, T_3 represent the high dose group of fermentation products, red represents the up-regulation, blue represents the downregulation.
